# Nitrogen Availability and Changes in Precipitation Alter Microbially Mediated NO and N_2_O Emissions From a Pinyon–Juniper Dryland

**DOI:** 10.1111/gcb.70159

**Published:** 2025-03-27

**Authors:** Sharon Zhao, Alexander H. Krichels, Elizah Z. Stephens, Anthony D. Calma, Emma L. Aronson, G. Darrel Jenerette, Marko J. Spasojevic, Joshua P. Schimel, Erin J. Hanan, Peter M. Homyak

**Affiliations:** ^1^ Department of Environmental Sciences University of California Riverside California USA; ^2^ USDA Forest Service Rocky Mountain Research Station Albuquerque New Mexico USA; ^3^ Department of Microbiology and Plant Pathology University of California Riverside California USA; ^4^ Department of Botany and Plant Sciences University of California Riverside California USA; ^5^ Center for Conservation Biology University of California Riverside California USA; ^6^ Department of Evolution, Ecology, and Organismal Biology University of California Riverside California USA; ^7^ Environmental Dynamics and GeoEcology Institute University of California Riverside Riverside California USA; ^8^ Department of Ecology, Evolution and Marine Biology University of California Santa Barbara California USA; ^9^ Department of Natural Resources & Environmental Science University of Nevada Reno Nevada USA

**Keywords:** altered precipitation, ammonia‐oxidizing archaea, ammonia‐oxidizing bacteria, climate change, nitrification, nitrogen cycling

## Abstract

Climate change is altering precipitation regimes that control nitrogen (N) cycling in terrestrial ecosystems. In ecosystems exposed to frequent drought, N can accumulate in soils as they dry, stimulating the emission of both nitric oxide (NO; an air pollutant at high concentrations) and nitrous oxide (N_2_O; a powerful greenhouse gas) when the dry soils wet up. Because changes in both N availability and soil moisture can alter the capacity of nitrifying organisms such as ammonia‐oxidizing bacteria (AOB) and archaea (AOA) to process N and emit N gases, predicting whether shifts in precipitation may alter NO and N_2_O emissions requires understanding how both AOA and AOB may respond. Thus, we ask: How does altering summer and winter precipitation affect nitrifier‐derived N trace gas emissions in a dryland ecosystem? To answer this question, we manipulated summer and winter precipitation and measured AOA‐ and AOB‐derived N trace gas emissions, AOA and AOB abundance, and soil N concentrations. We found that excluding summer precipitation increased AOB‐derived NO emissions, consistent with the increase in soil N availability, and that increasing summer precipitation amount promoted AOB activity. Excluding precipitation in the winter (the most extreme water limitation we imposed) did not alter nitrifier‐derived NO emissions despite N accumulating in soils. Instead, nitrate that accumulated under drought correlated with high N_2_O emission via denitrification upon wetting dry soils. Increases in the timing and intensity of precipitation that are forecasted under climate change may, therefore, influence the emission of N gases according to the magnitude and season during which the changes occur.

## Graphical abstract

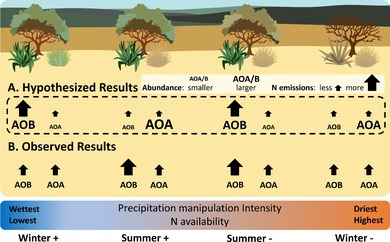

## 1 Introduction

Global changes in the timing and amount of precipitation can directly affect soil moisture, a key factor governing microbial processes responsible for nitrogen (N) cycling in terrestrial ecosystems (Ren et al. 2024; Vitousek et al. 2022; von Sperber et al. 2017). In dryland ecosystems, where potential evapotranspiration exceeds precipitation (precipitation: potential evapotranspiration ratio < 0.65; UNEP 1997; Cherlet et al. 2018), N can accumulate in soils because low rates of diffusion can constrain biological N uptake (Homyak et al. 2017). At the onset of winter rains, however, N that has accumulated over the dry season can become bioavailable to microbes and processed more quickly than plants can take it up (Davidson et al. 1991; Eberwein et al. 2020; Homyak et al. 2016; Krichels et al. 2023a). Thus, rapid microbial N processing can result in large gaseous N losses even when primary productivity remains N limited (Homyak et al. 2014; Osborne et al. 2022). When these gaseous N losses occur, they are often dominated by nitric oxide (NO), an air pollutant at high concentrations, and nitrous oxide (N_2_O), a powerful greenhouse gas and driver of stratospheric ozone destruction (Ravishankara et al. 2009; Sha et al. 2021; Tian et al. 2020).

Soil emissions of NO and N_2_O, produced primarily by nitrification and denitrification, account for up to one‐third of global N_2_O emissions (Davidson and Kanter 2014), and over 70% of estimated terrestrial NO emissions, with some of the highest emissions measured in drylands (Davidson and Kingerlee 1997). Nitrification is an aerobic process where ammonia (NH_3_; measured as NH_4_^+^ in soils) is oxidized by nitrifiers to nitrate (NO_3_^−^), whereas denitrification is an anaerobic process where NO_3_^−^ is used as an alternative electron acceptor and reduced to NO, N_2_O, and dinitrogen gas (N_2_; Firestone and Davidson 1989). Nitrification may be an especially important mechanism of NO and N_2_O emissions in drylands because the soil saturation events that create the suboxic conditions needed for denitrification occur less frequently (Osborne et al. 2022). However, it is not clear how changes in precipitation patterns may affect the abundance of the two major groups of nitrifying microorganisms producing these trace gases: ammonia‐oxidizing bacteria (AOB) and ammonia‐oxidizing archaea (AOA). Given that AOB may emit more NO and N_2_O during nitrification than AOA (Mushinski et al. 2019; Prosser et al. 2019), understanding the controls over AOB nitrification in drylands (which cover approximately 40% of Earth's terrestrial surface; Cherlet et al. 2018) is necessary for developing accurate NO and N_2_O emission budgets globally.

AOA and AOB emit NO and N_2_O at varying rates due to differences in their nitrification pathways (Banning et al. 2015; Fuchslueger et al. 2014; Koch et al. 2019; Prosser et al. 2019). Nitrification consists of several sequential steps, beginning with the oxidation of ammonia (NH_3_) to hydroxylamine (NH_2_OH), followed by the oxidation of NH_2_OH to nitrite (NO_2_^−^) through the pathway of NO (Caranto and Lancaster 2017), and ending with the production of NO_2_^−^ and nitrate (NO_3_^−^) (Heil et al. 2016). While AOA and AOB have similar ammonia‐oxidizing enzymes (ammonia‐monooxygenase, or AMO), differences in NH_2_OH oxidation pathways may allow more NO/N_2_O to escape to the atmosphere during AOB nitrification relative to AOA (Stein 2019). This is because AOA likely require NO as a coreactant during the oxidation of NH_2_OH to NO_2_^−^, which may constrain the loss of NO (Kozlowski et al. 2016; Prosser et al. 2019). Furthermore, AOB can enzymatically reduce NO to form N_2_O during nitrification, whereas there is no evidence for a similar pathway in AOA (Hink et al. 2017; Prosser et al. 2019). However, both AOA and AOB can release nitrification intermediates (NH_2_OH and NO_2_^−^) into the soil (Ermel et al. 2018), where denitrification, NH_2_OH decomposition, or chemodenitrification can transform them into NO and/or N_2_O (Firestone and Davidson 1989; Heil et al. 2016; Zhu‐Barker et al. 2015). Because nitrification is an important source of N trace gas emissions in drylands (Homyak et al. 2016; Krichels et al. 2022), soils with higher AOB than AOA activity may further amplify N losses from these ecosystems (Adair and Schwartz 2008; Prosser et al. 2019).

**FIGURE 1:**
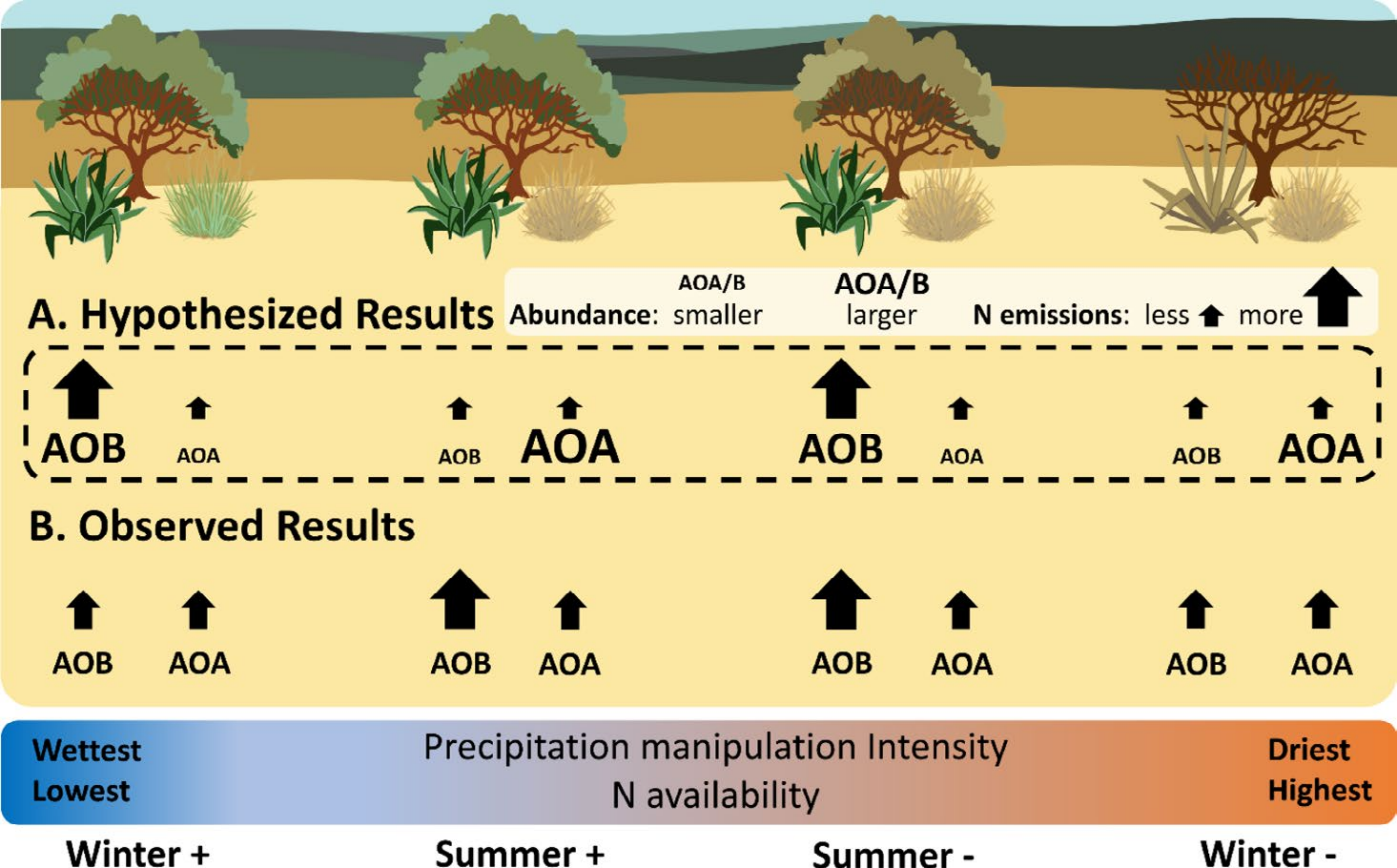
(A) Hypothesized effects of precipitation manipulation on nitrifier communities and NO emissions after wetting dry soils in the laboratory. We predicted that NO emissions after wetting dry soils would be highest in the summer rainfall exclusion treatment (*Summer‐*) and lower in the summer rainfall addition treatment (*Summer+*). We also predicted that altering winter precipitation would affect AOB nitrification regardless of soil N availability, lowering AOB‐derived NO and N_2_O emissions in the *Winter‐* treatment because of extreme drought, and increasing AOB‐derived NO and N_2_O emissions in the *Winter+* treatment because of higher microbial activity in moist soils. (B) Observed results after wetting dry soils in the laboratory. The size of the black arrows corresponds to process rates (NO emissions) in each of the field treatments and size of the text (AOA and AOB) represent nitrifier abundance in each of the field treatments. For the observed results, arrow and text size only differ among treatments if differences were statistically significant (*p* < 0.05).

The activity of AOA and AOB and related NO and N_2_O emissions may be affected by soil moisture and N availability, which are both influenced by the timing and amount of precipitation (Figure 1). For example, AOB may nitrify more than AOA when ammonium (NH_4_^+^) is abundant (Figure 1, *Summer‐*; Hink et al. 2017; Prosser et al. 2019), and such conditions may occur during dry periods when limited diffusion constrains N immobilization by microbes and plants, allowing NH_4_^+^ to accumulate (Homyak et al. 2017; Zhong et al. 2014). In contrast to dry periods, more frequent precipitation in drylands may favor plant primary productivity and N assimilation, lowering soil N availability while promoting efficient N recycling by AOA over AOB (Figure 1, *Summer+*). However, while high soil N availability may promote nitrification by AOB under dry conditions, increasingly dry conditions may push AOB nitrifiers past a “tipping point,” where they become limited by water (Elrys et al. 2024). AOA may be more tolerant of extreme dry conditions (Adair and Schwartz 2008; Banning et al. 2015; Zhang et al. 2012) and become more abundant than AOB in hyperarid soils (Delgado‐Baquerizo et al. 2013, 2016). If water limitation overrides N availability as the predominant control over AOB nitrification under dry conditions, then drier soils may favor drought‐tolerant AOA, potentially reducing overall NO and N_2_O emissions (Figure 1; *Winter‐*).

To understand how shifts in precipitation patterns may affect soil moisture and nitrifier‐derived NO and N_2_O emissions, we leveraged a dryland site that experiences two distinct plant growing seasons: a winter growing season characterized by cool temperatures and frequent precipitation that keeps soils relatively moist, and a summer growing season characterized by occasional monsoonal rains that infrequently interrupt otherwise hot and dry conditions (Ludwig et al. 1988; Spasojevic et al. 2022). Thus, while reductions in both winter and summer precipitation may reduce substrate diffusion and increase N concentrations, dry winter conditions would be expected to be relatively more extreme than dry summer conditions for nitrifying communities, as the wet season with high soil moisture would be key to generating nitrifier biomass reserves required to survive the dry summer (Bradford et al. 2020; Collins et al. 2008; Krichels et al. 2023b). In contrast, reducing summer precipitation may not induce additional water stress on nitrifier communities that are already adapted to hot and dry summers (Adair and Schwartz 2008; Banning et al. 2015; Delgado‐Baquerizo et al. 2016). Similarly, adding extra water during the winter may allow nitrifiers to generate larger biomass reserves to endure the summer, whereas adding extra water during summers may not appreciably affect soil moisture due to high temperatures and evaporative demand that maintain dry conditions. Summer and winter precipitation may, therefore, have distinct effects on soil N availability and nitrifier activity, leading us to ask: How do shifts in summer and winter precipitation affect nitrifier‐derived N trace gas emissions?

To answer this question, we manipulated both summer and winter precipitation in a Pinyon‐Juniper dryland; we excluded all summer or winter precipitation and added the collected precipitation to adjacent plots to increase precipitation amount by an average of 32%. We then measured NO and N_2_O emissions after selectively inhibiting AOA and AOB nitrification in soils collected from all plots. We hypothesized that changes in soil N availability would control AOB‐derived N trace gas emissions under shifts in summer precipitation that may not be extreme enough to induce water limitation of AOB, but that alter soil N availability. From this hypothesis, we predicted that NO and N_2_O emissions following rewetting would be higher in the summer rainfall exclusion treatment (Figure 1; Su*mmer‐*) and lower in the summer rainfall addition treatment (Figure 1; *Summer+*). We also hypothesized that more extreme water stress would limit the activity of AOB relative to the more drought‐tolerant AOA under shifts in winter precipitation, lowering total NO and N_2_O emissions because AOB may produce more NO and N_2_O than AOA. We predicted that altering winter precipitation would affect AOB nitrification regardless of soil N availability, lowering AOB‐derived NO and N_2_O emissions in the *Winter‐* treatment because of more extreme water stress, and increasing AOB‐derived NO and N_2_O emissions in the *Winter+* treatment because of higher microbial activity in moist soils (Figure 1).

## 2 Methods & Materials

### 2.1 Field Site Description

Our study was conducted in Pinyon Flats, part of the Boyd Deep Canyon Reserve (33°36′36.7“N, 116°27′06.1“W) in Southern California. This field site has two distinct plant growing seasons: winter (November–May) and summer (June–October). Winters are cool and receive most of the year's precipitation (in an average precipitation year), while summers are warm and dry; however, monsoonal precipitation promotes plant growth in summer despite soils drying quickly due to high evaporative demand (Spasojevic et al. 2022; Figures S1–S2; Pinyon Crest weather data are publicly available at https://deepcanyon.ucnrs.org/weather‐data/). Winters between 2000 and 2022 had a mean monthly minimum temperature of 7.6°C, a mean monthly maximum temperature of 19.2°C, and a mean monthly precipitation of 22.4 mm. Summers between 2000 and 2022 had a mean monthly low temperature of 20.5°C, a mean monthly high temperature of 33.2°C, and a mean monthly precipitation of 16.3 mm.

Soils at the site have a gravelly fine sandy loam texture, are mapped in the Omstott series, and are classified as loamy, mixed, nonacidic, mesic, shallow Typic Xerorthents (Web Soil Survey. Available at https://websoilsurvey.nrcs.usda.gov/app/WebSoilSurvey.aspx). The mean total C content (0–10 cm depth) is 0.48% ± 0.12% and the mean total N content is 0.03% ± 0.01% with a soil pH of 7.6 ± 0.6 (Krichels et al. 2023b). In 2023, soil pH was measured in September and was7.7 ± 0.10. Historically, vegetation was dominated by *Juniperus californica* and *Pinus monophylla* with many herbaceous plants growing in the interspaces between these two dominant species. However, *Juniperus californica* and *Pinus monophylla* have not reestablished dominance since the site burned in 1994 (Spasojevic et al. 2022).

### 2.2 Field Experimental Design and Soil Sampling

**FIGURE 2:**
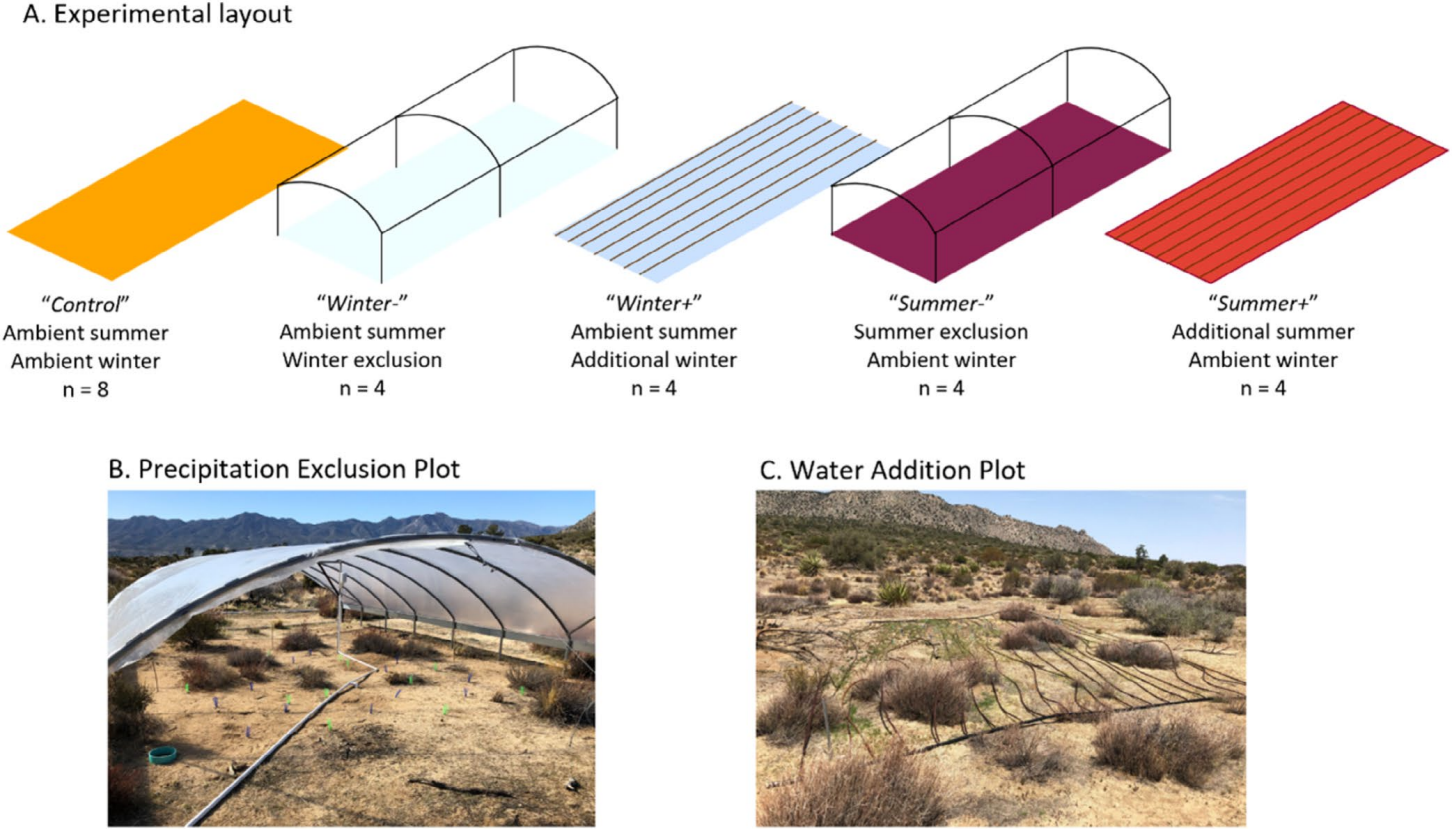
(A) Field experimental layout of our research site (adapted from Krichels et al. 2023b). (B) Plastic roofing was used to exclude precipitation in *Winter‐* and *Summer‐* plots and (C) irrigation lines to add precipitation to *Winter+* and *Summer+* plots.

In July 2018, we established 24 plots (6 × 8.5‐m) and began manipulating precipitation either by excluding natural precipitation or by adding water during the winter and summer seasons (increasing seasonal precipitation by an average of 32%; Figure 2). Approximately 335 mm of precipitation was excluded from *Winter‐* plots (*n* = 4) from November 8, 2018–July 23, 2019, 252 mm from October 22, 2019–June 18, 2020, and 85 mm from October 7, 2020–June 1, 2021. Approximately 73 mm of precipitation was excluded from *Summer‐* plots (*n* = 4) from July 23, 2019–October 22, 2019, 0 mm from June 18, 2020–October 7, 2020, and 57 mm from June 1, 2021–October 7, 2021. Extra water was added to *Winter +* plots (*n* = 4; 132 mm in 2019, 106 mm in 2020, 7.3 mm in 2021) and *Summer +* plots (*n* = 4; 29 mm in 2018, 22 mm in 2019, 0 mm in 2020, and 15 mm in 2021). All precipitation data are summarized in Table S1. The extra water was added within two weeks of measurable precipitation. *Control* plots (*n* = 8) received ambient precipitation in both summer and winter (194 mm annual precipitation in 2018, 400 mm in 2019, 189 mm in 2020, 185 mm in 2021). *Winter‐* and *Summer‐* exclusion plots were sheltered with metal frames and polyethylene plastic (Tuff Lite IV 28×70 ft. TES IR/AC, Berry Plastics, Evansville, IN) to exclude precipitation. The shelter did not fully protect plots from rainfall blown in by heavy winds or surface water runoff. *Summer‐*coverings were installed in early summer (June/July) and kept on until the onset of rainfall during the winter wet season (October/November). Precipitation was collected from the exclusion shelters using a downslope 102‐mm‐diameter PVC system connected to four 5.7 m^3^ water tanks. *Winter +* and *Summer +* addition plots were irrigated with the collected precipitation. Water from the water tanks was pumped and distributed evenly to the plots using 17‐mm‐diameter drip tubing (Netafim, Tel Aviv, Israel), and the amount of water was validated using water meters (DAE Controls, Sterling Heights, MI; model 1 DAE AS250U).

Soil samples from the A horizon (0–10 cm depth; ~500 g) from each of the 24 plots were collected in October 2021, corresponding to the end of the dry season during late summer at our site. Soils were brought to the lab and air‐dried at room temperature for approximately one month before sieving (2 mm). We determined soil water holding capacity (WHC) for each soil sample as the water retained by water‐saturated soils against gravity for an 8‐h period (soils were held inside an air‐tight container to minimize evaporative water losses). While laboratory incubations can disturb the complex in situ environmental conditions that affect soil N cycling, including the disruption of biological soil crusts that fix N (Belnap and Lange 2003) and aggregates that harbor anoxic environments that can promote denitrification (Sexstone et al. 1985; Schlüter et al. 2024), these controlled environments are required to evaluate the potential contribution of AOA and AOB communities to N trace gas emissions and, thereby, improve mechanistic understandings of soil processes.

### 2.3 Assessing AOA and AOB Activity in the Laboratory

Soil samples from each of the 24 plots collected in October 2021 were divided into four 50‐g subsamples, with each subsample added to a 118‐mL glass canning jar assigned to one of the following four treatments: 100% WHC Control, 50% WHC Control, 50% WHC AOB inhibition, and 50% WHC total nitrifier inhibition (i.e., inhibition of both AOA and AOB). Based on our design with 24 plots under rainfall manipulation (*n* = 8 *Control*, *n* = 4 each for *Winter‐*, *Winter+*, *Summer‐*, and *Summer+*) and four WHC/inhibition incubations per plot, we monitored N dynamics in 96 soil incubations. We used 50% WHC to stimulate nitrification (Pilegaard 2013) and 100% WHC to stimulate denitrification and N_2_O production (Firestone and Davidson 1989). We did not measure the relative contribution of AOA‐ and AOB nitrification to N trace gas emissions in the soils held at 100% WHC because we expected denitrification would dominate emissions at that water content (Firestone and Davidson 1989). After wetting each subsample to the corresponding WHC, we measured NO and N_2_O emissions, net nitrification, and net NH_4_^+^ production rates over a 48‐h period. To inhibit the oxidation of ammonia by AOB or by all nitrifiers, jar headspace was pre‐incubated for 24 h with either 1‐octyne (4 μmol L^−1^) to selectively inactivate AOB nitrification, or acetylene (6 μmol L^−1^) to selectively inactivate both AOA and AOB nitrification, following established methods for laboratory microcosm incubations (Taylor et al. 2013; Mushinski et al. 2019). While both 1‐octyne and acetylene work by inactivating the ammonia monooxygenase enzyme (AMO), 1‐octyne is specific to AMO enzymes from bacteria (Taylor et al. 2013). To ensure that no new AOB grew when soils were removed from the 1‐octyne headspace, soils were wet with a ~13‐mL solution (enough to reach 50% WHC) containing the bacterial growth inhibitor kanamycin (220 μg g^−1^ soil) at the beginning of each gas measurement (Mushinski et al. 2019). To reduce autotrophic nitrification activity, soils were wet with a solution containing kanamycin (220 μg g^−1^ soil), fusidic acid (an archaeal protein synthesis inhibitor; 800 μg g^−1^ soil), and nitrapyrin (total autotrophic nitrification inhibitor; 200 μg g^−1^ soil) according to Mushinski et al. (2019).

We measured soil extractable inorganic N (NH_4_^+^ and NO_3_^−^) before and after gas measurements to measure net rates of nitrification (measured as a change in NO_3_^−^ over the incubation), net N mineralization (measured as a change in both NH_4_^+^ and NO_3_^−^ over the incubation), and NH_4_^+^ production (measured as a change in NH_4_^+^ over the incubation). This was done by extracting 3 g of soil in 30 mL of 2 M KCl before wetting and after the 42‐h laboratory incubation. Soil samples were then shaken for one hour before being gravity filtered (Whatman 42 filter paper; 2.5 μm pore size) and frozen at 0°C until analysis. Extracts were analyzed using a colorimetric assay to measure soil extractable NO_3_^−^ + NO_2_^−^ (SEAL method EPA‐126‐A) and NH_4_^+^(SEAL method EPA‐129‐A) on a discrete analyzer (Seal AQ2, Mequon, Wisconsin, USA) in the Environmental Sciences Research Laboratory (https://envisci.ucr.edu/research/environmental‐sciences‐research‐laboratory‐esrl) at the University of California, Riverside. To determine net rates of nitrification, net N mineralization, and net NH_4_^+^ production, substrate concentrations measured at the initial time point (air‐dried soil prior to the start of the incubation) were subtracted from concentrations measured at the final time points (post incubation) and divided by the duration of the incubation (42 h).

### 2.4 NO and N_2_O Flux Measurements

Fluxes of NO and N_2_O were measured in microcosms coupled to a multiplexer (LI‐8150, LI‐COR Biosciences, Lincoln, Nebraska, USA) to route air to a chemiluminescent NO_2_ analyzer (Scintrex LMA‐3), an infrared gas CO_2_ analyzer (LI‐8100), and an N_2_O analyzer (Model#: 914–0060–0000‐0000; Los Gatos Research, ABB Inc., Quebec, Canada). The chemiluminescent NO_2_ analyzer was equipped with an in‐line CrO_3_ NO oxidizer (Drummond Technology Inc., Ontario, Canada) to convert NO to NO_2_. To calibrate the chemiluminescent NO_2_ analyzer, we made a standard curve by mixing an NO standard (0.0988 ppmv NO in N_2_ gas, Airgas) with zero‐grade air before measurements. The chemiluminescent NO_2_ analyzer was connected to the sample loop only during the first 10 min of each measurement. During this time, it sampled air at a rate of ~0.9 L min^−1^. Since this chemiluminescent analyzer consumes NO in the process of measuring it, the instrument vented air to the atmosphere and was not recirculated into the jars. To avoid negative pressure in the sample loop, zero air was introduced to the sample loop at the same rate of exhaust from the NO analyzer (~0.9 L min^−1^). We calculated NO emissions based on the difference between the inlet NO concentration and the measured outlet NO concentration at the end of the 10‐min incubation (Hall et al. 2018); preliminary tests confirmed that 10 min was enough time for the outlet NO concentration to reach equilibrium. After the 10‐min NO measurement, an automated three‐way valve was used to cut off both the NO analyzer and the zero air from the sample loop. For the next 5 min, gas was recirculated through the closed sample loop, and N_2_O emissions were calculated as the linear change in N_2_O concentration for each incubation using a custom R script (Andrews and Krichels 2022). We flushed the instrument loop for over one minute with laboratrory air between measurements at a rate of 1.5 L min^−1^. Instrument errors caused the loss of two of the total nitrifier inhibition samples from the *Summer+*, *Winter‐*, and *Winter +* treatments.

The contribution of AOA to NO emissions was calculated by subtracting the NO emitted in the total inhibition treatment from the NO emitted in the AOB inhibition treatment. The contribution of AOB to NO emissions was calculated by subtracting the NO emitted in the AOB inhibition treatment from the NO emitted in control soils wetted with water only.

### 2.5 Quantitative PCR (qPCR) of amoA

Microbial DNA was extracted using a DNA extraction kit (DNeasy PowerSoil Pro kit, Qiagen). Before proceeding with manufacturer instructions, we pre‐incubated 250 mg of soil overnight with 700 μL CD1 and 100 μL ATL at 4°C. To estimate the abundance of AOA and AOB, we used qPCR to measure the number of *amoA* gene copies from bacteria (using the AmoA1F/amoA2R primer set; Rotthauwe et al. 1997) and archaea (using the Arch‐amoAF/amoA2R primer set; Francis et al. 2005). The qPCR consisted of qPCR master mix (Forget‐Me‐Know EvaGreen, Biotium Inc., Fremon, CA), 2 mM MgCl_2_, 0.5 mg ml^−1^ BSA, 0.25 μM forward and reverse primer, H_2_O, and sample DNA; the reactions were run using the protocol described by Eberwein et al. (2020). Bacterial (*amo*A gene of *Nitrosomonas europaea* ATCC 19718) and archaeal (crenarchaeota genomic fragment 54d9) *amoA* sequences were used as standards. Standard curves were prepared using serial dilutions for both archaeal amoA (10^7^–10^3^ copies) and bacterial amoA (10^6^–10^2^ copies). The archaeal *amoA* standards had efficiencies of 87.3% (*R* ^2^ = 0.994), and bacterial *amoA* standards had efficiencies of 74.5% (*R* ^2^ = 0.993). We measured *amoA* gene abundance because it is more likely to reflect long term changes to nitrifier communities from seasonal precipitation manipulations compared to transcript abundance (Kunadiya et al. 2020; Orellana et al. 2019).

### 2.6 Statistical Analyses

Statistical analyses and figures were done on R version 4.1.2 (R Core Team 2023). We used analysis of variance (ANOVA) to test for significant differences between our variables, with field treatment as the independent variable and either cumulative NO and N_2_O over the duration of the incubation, NO emitted by AOA, NO emitted by AOB, or soil extractable NH_4_^+^ and NO_3_^−^ as the dependent variables. Separate models were run for each laboratory treatment (i.e., AOB inhibition, total inhibition, control). Model residuals were assessed for normality using a Shapiro–Wilk test (“olsrr” package in R) (Hebbali 2020). We log‐transformed cumulative NO emissions, cumulative N_2_O emissions, and initial nitrate concentrations so that model residuals more closely followed a normal distribution. If the ANOVA was significant, we then compared each treatment to the control using a Dunnett's test (“emmeans” package) (Lenth 2016).

We used multiple linear regression (lm function in R) to assess whether AOB or AOA‐derived NO emissions were associated with NH_4_^+^ availability, nitrifier copy number (AOB for AOB‐derived emissions and AOA for AOA‐derived emissions), or pH. We ran a similar model to assess whether total N_2_O emissions from the 100% WHC treatment were associated with NO_3_^−^ concentrations or soil pH. To determine which predictor variables were significant, we compared all possible models after dropping each fixed term with an *F*‐test (drop1 function in R) (R Core Team 2023; Tredennick et al. 2021).

## 3 Results

### 3.1 Soil NO and N_2_O Emissions in Laboratory Incubations

**FIGURE 3:**
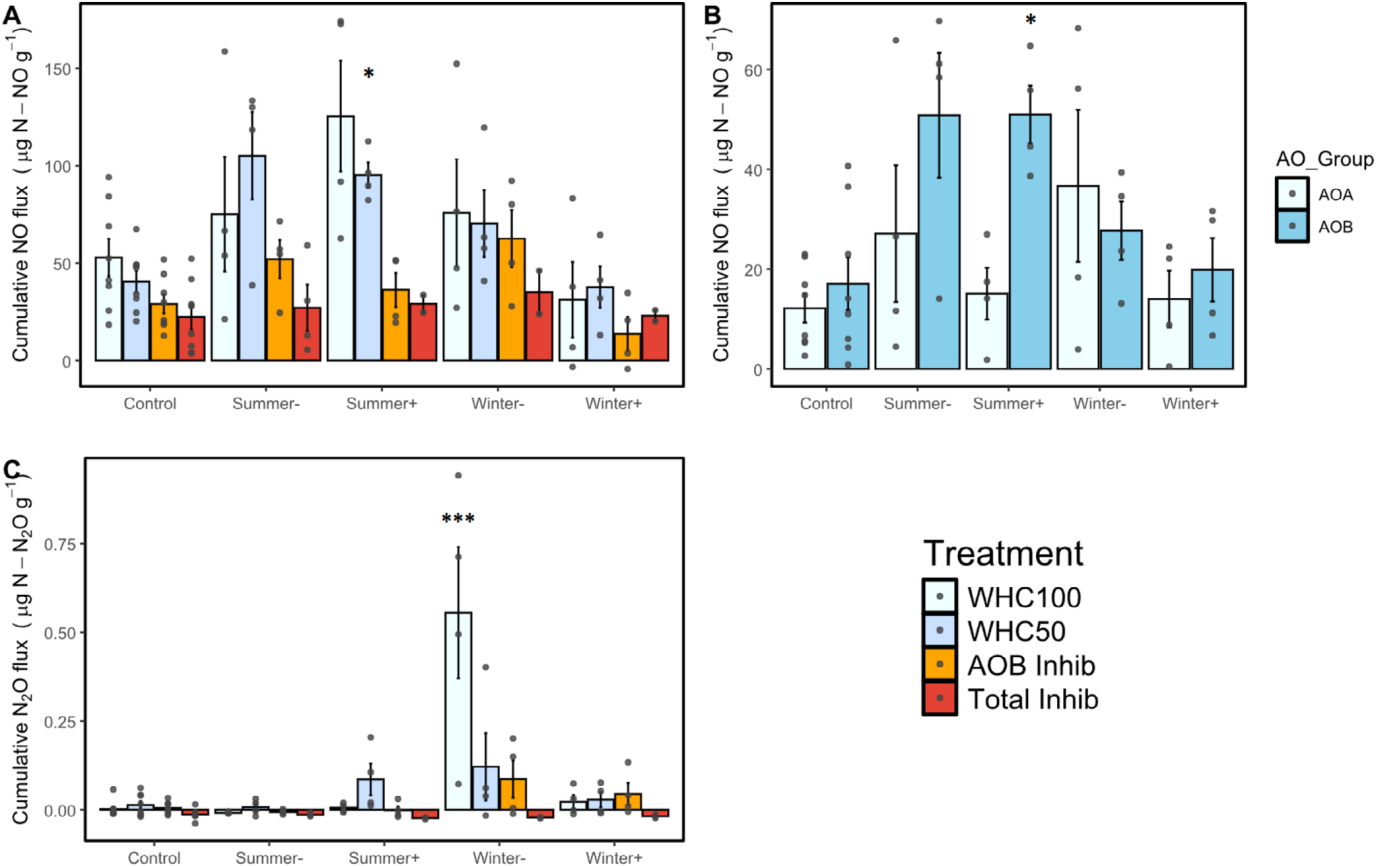
(A) Cumulative NO and (C) N_2_O emissions from 42‐h laboratory soil incubations held at different water holding capacities and, in the presence, or absence of nitrification inhibitors. WHC100 = 100% soil water holding capacity (light blue), WHC50 = 50% soil water holding capacity (blue), AOB Inhib = inhibition of AOB nitrification held at 50% soil water holding capacity (orange), Total Inhib = inhibition of both AOA and AOB nitrification held at 50% water holding capacity (red). (B) Cumulative NO flux emissions released by either the AOA or the AOB community. AOA group values were calculated by AOB inhibition flux—Total inhibition flux = AOA group flux. AOB group values were calculated by Control flux—Total inhibition flux—AOA flux = AOB group flux. Bars represent the average of individual observations represented by dots. Error bars represent the standard error (*n* = 8 for Control and *n* = 4 for *Summer+*, *Winter‐*, *Winter+*). Asterisks indicate treatments are significantly different from the control (*p < 0.05, **p < 0.01, ***p < 0.001). Due to instrument errors, *n* = 2 in the total inhibition treatment for *Summer+*, *Winter‐*, and *Winter+* plots.

Cumulative NO emissions from soils incubated in the laboratory at 50% WHC without inhibitors differed between summer and winter precipitation manipulations (WHC50; *F*_4,19_ = 6.54, *p* < 0.001; Figure 3A). Specifically, imposing extreme shifts in precipitation by either adding or excluding precipitation during the winter preceding our soil collection did not affect NO emissions; neither the *Winter* + (38 ± 11 μg NO‐N g soil^−1^; all data are presented as mean ± standard error) nor *Winter‐* (70 ± 17 μg NO‐N g soil^−1^) differed from the *Control* (40 ± 5 μg NO‐N g soil^−1^, *p* > 0.2; Figure 3A). However, moderately increasing precipitation during summer increased NO emissions; NO emissions from the *Summer* + treatment (95 ± 6 μg NO‐N g soil^−1^; *p* = 0.01) were significantly higher than the *Control* (41 ± 6 μg NO‐N g soil^−1^; Figure 3A). Moderately reducing precipitation (*Summer‐*) did not produce a statistically significant effect, yet NO emissions from the *Summer*‐ (105 ± 22 μg NO‐N g soil^−1^) were on average higher than the *Control* (*p* = 0.2).

AOB‐derived NO emissions measured in laboratory incubations held at 50% WHC differed among field treatments and mirrored cumulative soil NO emission trends (*F*_*4,19*_ = 5.27, *p* = 0.005; Figure 3B). Imposing extreme shifts in precipitation by either excluding or adding precipitation during the winter preceding soil collection did not affect AOB‐derived NO emissions; the *Winter‐* and *Winter +* treatments did not differ from the *Control* (*p* > 0.1) and accounted for less than 28% of the total cumulative NO emitted. However, moderately increasing precipitation during summer increased AOB‐derived NO emissions; emissions were higher in the S*ummer‐* (50.8 ± 10.8 μg NO‐N g soil^−1^; *p* = 0.04) and *Summer +* treatments (51.0 ± 5.0 μg NO‐N g soil^−1^) than in the *Control* (17.1 ± 4.9 μg NO‐N g soil^−1^), though for *Summer +* the difference was only significant at *p* = 0.06. AOB‐derived NO emissions accounted for over 50% of the cumulative NO emitted in both the *Summer‐* and *Summer +* treatments.

Imposing extreme or moderate shifts in precipitation by either adding or excluding precipitation in the winter or summer did not affect AOA‐derived NO emissions measured in the lab at 50% WHC, averaging 19.5 ± 3.8 μg NO‐N g soil^−1^ across all treatments (*F*_*4,19*_ = 1.56, *p* = 0.23; Figure 3B). AOA‐derived NO emissions accounted for less than 38% of the cumulative NO emissions from all treatments.

Imposing extreme or moderate shifts in precipitation did not affect N_2_O emissions from soils incubated at 50% WHC and were always less than 0.15 μg N_2_O‐N g soil^−1^ (WHC50; *F*_*4,19*_ = 1.45, *p* = 0.25; Figure 3C). However, N_2_O emissions measured in 100% WHC incubations without inhibitors differed among field treatments (*F*_*4,19*_ = 8.8, *p* < 0.001) and were highest from soils exposed to extreme precipitation reduction (*Winter‐* plots), averaging 0.58 ± 0.20 μg N_2_O‐N g soil^−1^ relative to just 0.0003 ± 0.008 μg N_2_O‐N g soil^−1^ measured in the *Control* (*p* < 0.001). We note that these high N_2_O emissions occurred exclusively in plots exposed to extreme precipitation reduction (*Winter‐*) and incubated at 100% WHC when both soil extractable NO_3_^−^ was higher and net N mineralization and nitrification rates were lower than the *Control* (see section 3.2).

### 3.2 Field Soil Water Content and Extractable Inorganic N

Soil moisture (measured as gravimetric water content) differed among the precipitation manipulation treatments (*F*_*4,19*_ = 49, *p* < 0.001; Figure S4). Soil moisture was lower in the treatments where we excluded summer precipitation (*Summer‐* treatment; 0.009 ± 0.007 g water g soil^−1^) than in the *Control* (3.7 ± 0.18 g water g soil^−1^; *p* < 0.001), but no other treatments differed from the *Control* (*p* > 0.1). The *Winter‐* and *Winter +* treatments extended until June 2021, suggesting that by the time we collected soil samples at the end of summer, October 2021, soil moisture had already equilibrated with *Control* plots.

**FIGURE 4:**
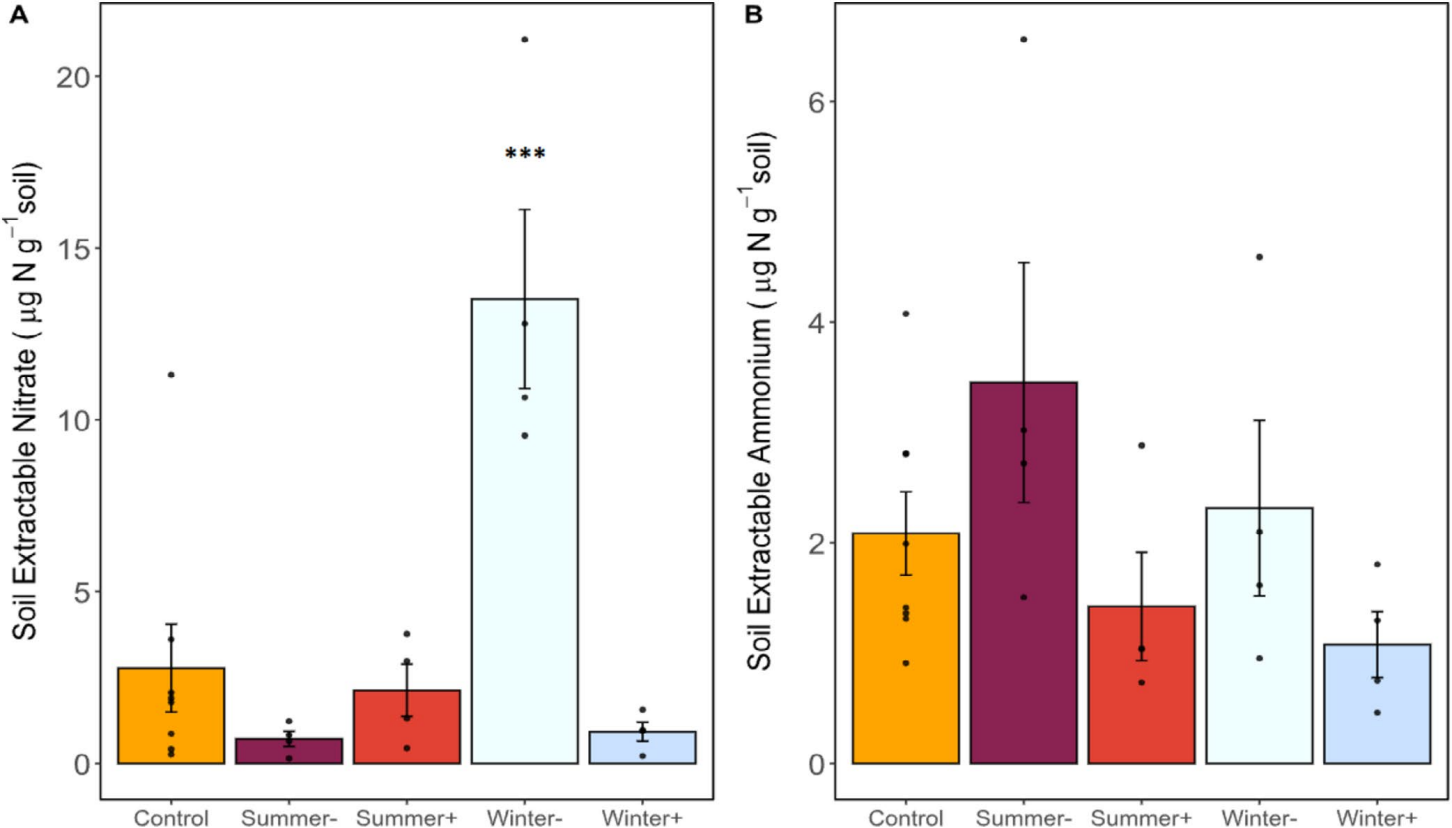
Soil extractable (A) nitrate (NO_3_^−^) and (B) ammonium (NH_4_^+^) concentrations from field‐collected soils prior to laboratory incubations. Bars represent the average of individual observations represented by black dots. Error bars represent standard errors (*n* = 4, except for control treatments where *n* = 8). Asterisks indicate treatments are significantly different than the control (***p < 0.001). For a description of rainfall manipulation treatments see Figure 1.

Soil extractable NO_3_^−^ varied in response to manipulating field precipitation (*F*_*4,19*_ = 6.3, *p* = 0.002), but NH_4_^+^ did not (*F*_*4,19*_ = 1.9, *p* = 0.2; Figure 4). Excluding precipitation in winter promoted the highest accumulation of NO_3_^−^; NO_3_^−^ was higher in the *Winter‐* treatment (13.5 ± 2.6 μg NO_3_^−^‐N g^−1^ soil; *p* = 0.008) than in the *Control* (2.8 ± 1.3 μg NO_3_^−^‐N g^−1^ soil; Figure 4A). In contrast to excluding precipitation, adding precipitation in the winter limited the accumulation of N in soils; the *Winter +* treatment had the lowest average NO_3_^−^ (0.92 ± 0.28 μg NH_4_^+^‐N g^−1^) and NH_4_^+^ (1.08 ± 0.30 μg NH_4_^+^‐N g^−1^) but did not differ significantly from the *Control* (2.8 ± 1.3 μg NO_3_^−^‐N g^−1^, *p* = 0.64; 2.1 ± 0.38 μg NH_4_^+^‐N g^−1^, *p* = 0.59).

### 3.3 Net N Mineralization, Nitrification, and NH_4_ ^+^ Production in Laboratory Incubations

**FIGURE 5:**
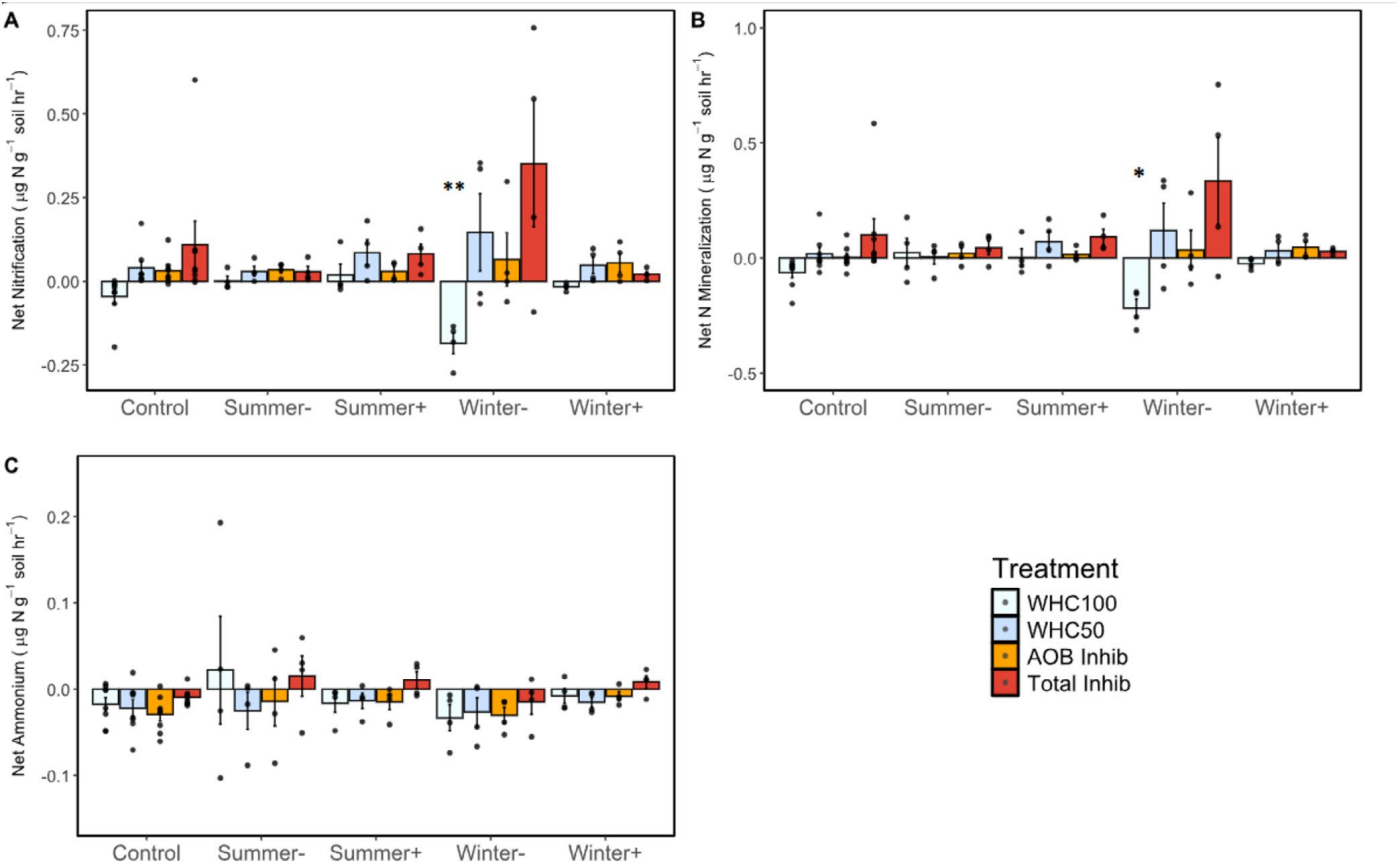
(A) Net nitrification, (B) net N mineralization, and (C) net NH_4_^+^ production rates from 42‐h laboratory soil incubations held at different water holding capacities and in the presence or absence of nitrification inhibitors. WHC100 = 100% soil water holding capacity (light blue), WHC50 = 50% soil water holding capacity (blue), AOB Inhib = inhibition of AOB nitrification held at 50% soil water holding capacity (orange), Total Inhib = inhibition of both AOA and AOB nitrification held at 50% water holding capacity (red). Error bars represent standard errors (*n* = 4, except for the Control where *n* = 8) and dots show individual measurements. Asterisks indicate treatments are significantly different from the control (*p < 0.05, **p < 0.01). For a description of rainfall manipulation treatments see Figure 1.

Manipulating precipitation did not affect net nitrification rates when soils were incubated for 42 h at 50% WHC whether we used AOB inhibitors (*F*_*4,19*_ = 0.21, *p* = 0.93), both AOA and AOB inhibitors (*F*_*4,19*_ = 1.9, *p* = 0.15; Figure 5A), or no inhibitors (*F*_*4,19*_ = 0.45, *p* = 0.77). However, when soils were incubated at 100% WHC after excluding winter precipitation (*Winter‐*), net nitrification rates decreased (*F*_*4,19*_ = 8.8, *p* < 0.001); net nitrification rates were lower in soils from the *Winter‐* treatment (−0.18 ± 0.03 μg N g^−1^ h^−1^) than in the *Control* (−0.046 ± 0.02 μg N g^−1^ h^−1^).

As with net nitrification, manipulating precipitation did not affect net N mineralization rates when soils were incubated for 42 h at 50% WHC whether we used AOB inhibitors (*F*_*4,19*_ = 0.24, *p* = 0.91), both AOA and AOB inhibitors (*F*_*4,19*_ = 1.6, *p* = 0.21; Figure 5B), or no inhibitors (*F*_*4,19*_ = 0.70, *p* = 0.61). When soils were incubated at 100% WHC (*F*_*4,19*_ = 8.83, *p* = 0.0003), net N mineralization rates were lower in the *Winter‐* treatment (−0.22 ± 0.04 μg N g^−1^ h^−1^; *p* = 0.002) than in the *Control* (−0.064 ± 0.022 μg N g^−1^ h^−1^).

Manipulating precipitation did not affect net NH_4_^+^ production rates when soils were incubated for 42 h at 50% WHC whether we used AOB inhibitors (*F*_*4,19*_ = 0.60; *p* = 0.29), both AOA and AOB inhibitors (total inhibition; *F*_*4,19*_ = 1.3, *p* = 0.29), or no inhibitors (*F*_*4,19*_ = 0.17, *p* = 0.95). Incubating soils at 100% WHC without inhibitors also had no effect on net NH_4_^+^ production rates (*F*_*4,19*_ = 58, *p* = 0.68; Figure 5C).

### 3.4 AOA and AOB Abundance

**FIGURE 6:**
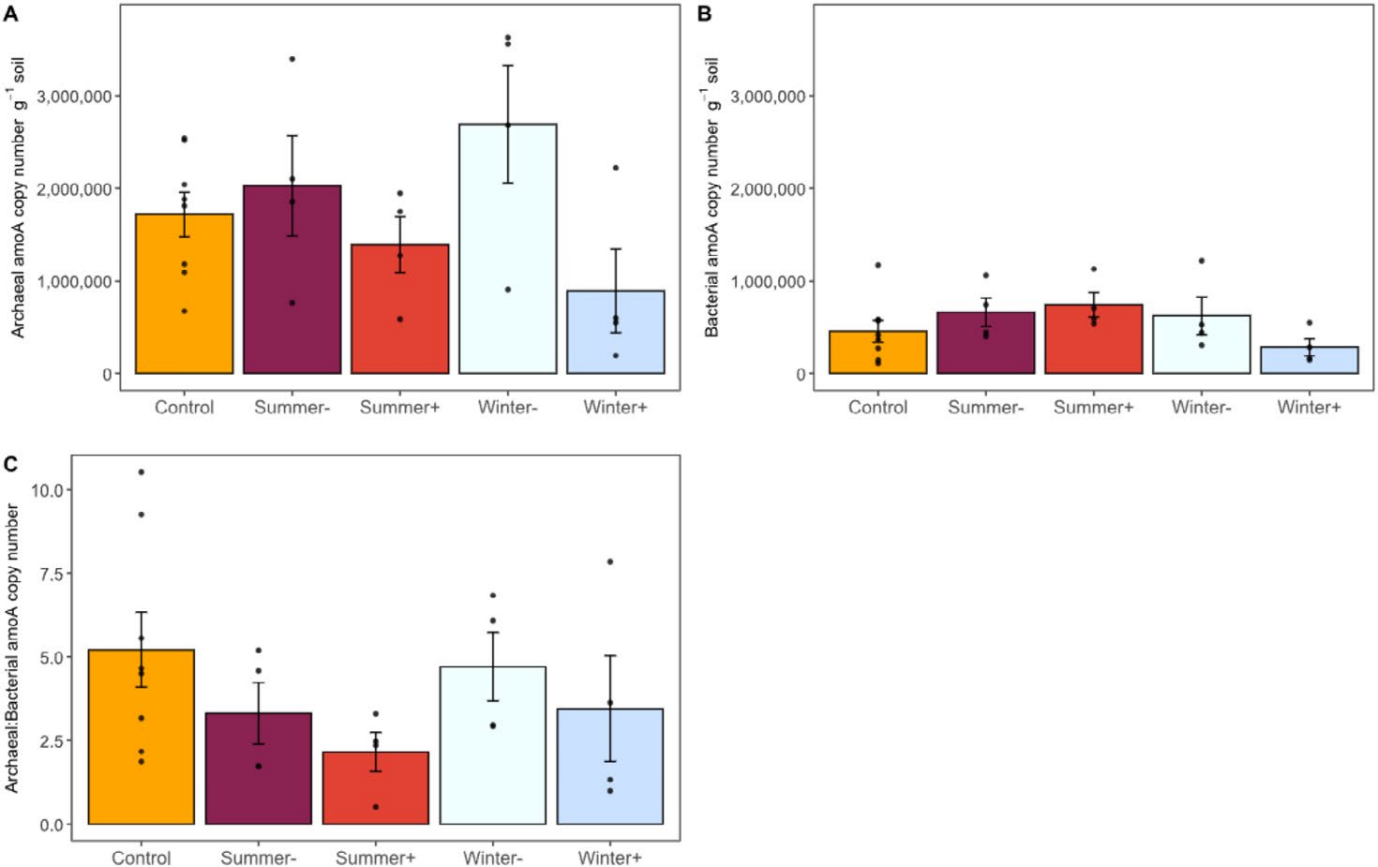
Field measurements of (A) archaeal amoA (AOA) gene copy number, (B) bacterial amoA (AOB) gene copy number and the (C) ratio of AOA:AOB gene copy numbers from field‐collected soils prior to laboratory incubations. Bars represent the average of individual observations represented by dots. Error bars represent standard errors (*n* = 4, except for control treatments where *n* = 8). For a description of rainfall manipulation treatments see Figure 1.

On average, we detected more *amoA* gene copies for AOA than AOB across soils collected from our experimental plots (Figure 6A,B). Manipulating precipitation had a marginal effect on AOA copy numbers (*F*_*4,19*_ = 2.3; *p* = 0.096), and there were 1.5× more AOA in the *Winter‐* treatment (2.7 × 10^6^ ± 6.3 × 10^5^ *amoA* gene copy numbers g^−1^ soil; *p* = 0.28) than in the *Control* (1.7 × 10^6^ ± 2.4 × 10^5^ copy numbers g^−1^ soil). Manipulating precipitation did not significantly affect AOB copy numbers (*F*_*4,19*_ = 1.4; *p* = 0.27; Figure 6B), and the AOA:AOB ratio did not differ between precipitation treatments (*F*_*4,19*_ = 1.14, *p* = 0.368; average AOA:AOB = 3.76 ± 0.48 copies across all field treatments; Figure 6C).

### 3.5 Relationships Between NO or N_2_O Emissions and N Availability, pH, or Nitrifier Abundance

AOB‐derived NO emissions were positively associated with AOB copy number (*p* = 0.02) but were not correlated with NH_4_^+^ availability or soil pH (*p* > 0.05; Figure S5). AOA‐derived NO emissions were not correlated with soil moisture, AOA copy number, NH_4_^+^ availability, or soil pH (*p* > 0.5; Figure S6). N_2_O emissions were positively correlated with soil NO_3_^−^ availability (*p* < 0.001; Figure S7).

## 4 Discussion

We manipulated the amount and timing of seasonal precipitation in the field and incubated soils in the laboratory to understand whether shifts in summer and winter precipitation could alter soil N pools and the ratio of AOA:AOB‐derived NO emissions in a pinyon‐juniper dryland. We found that altered seasonal precipitation patterns increased N loss via gaseous pathways at our dryland site, highlighting the potential role of climate change on limiting ecosystem N availability. Specifically, either adding or excluding precipitation during the summer dry season increased AOB‐derived NO emissions when we wetted dry soils at the end of summer. This suggests that moderately altering the length of time between storms during the dry season—consistent with projected increases in precipitation variability (Dai 2013; Bradford et al. 2020)—increased N losses via NO. In contrast to the dry season, altering precipitation during the winter wet season had no effects on NO emissions; however, the more extreme dry periods imposed by excluding wet season precipitation (*Winter‐*) favored the emission of N_2_O. Our observations suggest that altering summer precipitation in pinyon‐juniper drylands may stimulate NO emissions by increasing soil N availability, or by alleviating moisture limitations on nitrifier activity when dry soils are wetted. In the historically winter wet season, excluding precipitation may induce more severe water stress that limits nitrifier emissions while simultaneously stimulating denitrifier‐derived N_2_O emissions upon rewetting dry soils to amplify the emission of this powerful greenhouse gas.

### 4.1 Altering Summer Precipitation Increased Soil NO and N_2_O Emissions

Manipulating precipitation during the summer dry season affected AOB‐derived NO emissions, whether we added (*Summer+*) or excluded (*Summer‐*) precipitation. The *Summer‐* treatment was designed to exclude the few monsoonal summer rainfall events at our site (Figure S1), maintaining consistently dry conditions that we expected would increase NH_4_^+^ availability and give AOB a competitive advantage over AOA (Prosser et al. 2019). We found evidence in support of dry conditions increasing soil NH_4_^+^ availability despite our one‐time bulk soil extraction at the end of summer (Figure 4B) because: (i) background soil extractable NH_4_^+^ in the *Control* increased by ~6 times throughout the dry summer, reaching 0.80 ± 1.43 μg NH_4_^+^‐N g^−1^ in August 2021 (Figure S3), and (ii) we measured some of the highest extractable NH_4_^+^ concentrations in the *Summer‐* treatments where we intensified summer drought (Figure 4). Nevertheless, NH_4_^+^ availability was not associated with AOB‐derived NO emissions across all treatments, suggesting that NH_4_^+^ availability is not the primary control over AOB activity in our sites. Instead, moisture may have constrained AOB activity under the more extreme dry conditions (*Winter‐* treatment) despite high NH_4_^+^ availability, preventing a positive correlation between AOB‐derived NO emissions and NH_4_^+^ across all treatments. Given the higher AOB‐derived NO emissions in the moderately dry conditions in the *Summer‐* treatment, AOB may have remained active enough to nitrify some of the available NH_4_^+^ to NO, suggesting that NH_4_^+^ accumulation can stimulate NO production if moisture does not limit AOB activity.

Because the *Summer +* treatment was designed to decrease the amount of time between rain events and increase soil moisture, we expected plant and microbial N immobilization to lower NH_4_^+^ availability, thereby limiting NO emissions. However, NH_4_^+^ availability was not lower than the control, and NO emissions were instead higher from the *Summer +* treatment. Instead of lowering NH_4_^+^ availability, adding extra water may have helped sustain AOB activity during the dry season and favored NO emissions upon wetting dry soils in the lab. This interpretation is consistent with the “pulse‐reserve” paradigm, where organisms build biomass reserves during wet periods that allow them to respond more quickly to subsequent precipitation (Collins et al. 2008). Indeed, we observed a positive correlation between AOB abundance and AOB‐derived NO emissions, suggesting that NO emissions increase with AOB abundance. While AOB abundance and AOA:AOB *amoA* gene copy ratio did not differ among treatments, we observed the most AOB and the smallest AOA:AOB *amoA* gene copy ratio in the *Summer +* treatments (Figure 6C). Even though these changes in AOB abundance were not statistically significant, changes in soil moisture may affect AOB activity without changing their abundance, consistent with gene abundance often being a poor predictor of function (Rocca et al. 2015). Altogether, increased precipitation during the summer dry season may allow AOB to remain more active, favoring AOB‐derived NO emissions upon wetting dry soils.

### 4.2 Altering Winter Precipitation Increased N_2_O Emissions but Did Not Affect NO Emissions

Imposing more extreme shifts in precipitation by either adding (*Winter+*) or excluding (*Winter‐*) wet season precipitation had no effect on NO emissions. Not detecting a treatment effect on NO was consistent with not detecting treatment effects on processes known to influence NO production, such as net nitrification, net N mineralization, and net NH_4_^+^ production rates measured in lab incubations held at 50% WHC (Figure 5). Based on the *Winter +* treatment, these results suggest that winter wet season soil N cycling is not limited by water at our sites—soil moisture remains relatively high throughout the winter (Figure S1)—such that adding extra precipitation had no effects on AOB abundance, N availability, and NO or N_2_O emissions. Consistent with this finding, we have not yet detected a *Winter +* treatment effect on plant biomass at our site that could imply greater competition between plants and nitrifiers for NH_4_^+^ (Spasojevic et al. 2022), further helping to explain why AOB‐derived NO emissions from *Winter +* soils did not differ from the C*ontrol*. In contrast to the *Winter +* treatment, extreme water limitation imposed by the *Winter‐* treatment reduced plant biomass by up to 100% (Spasojevic et al. 2022), and may have allowed N to accumulate in soils in the absence of plant N assimilation—soil extractable NO_3_^−^ was higher in the *Winter‐* soils than in the *Control* (Figure 4) with NH_4_^+^ also being higher than the *Control* the prior summer and one month before we collected soils (September 2020 and August 2021; Figure S3). Yet, this increase in extractable N did not stimulate AOB‐derived NO emissions upon wetting dry soil, suggesting that water limitation may have become too extreme to support NO production by AOB (Adair and Schwartz 2008; Banning et al. 2015; Elrys et al. 2024; Zhang et al. 2012). Extreme dry periods during the historically wet winter growing season (i.e., the *Winter‐* treatment), followed by the summer dry season, may exceed a tipping point beyond which AOB activity is limited by water stress.

While AOB‐derived NO emissions were larger than those from AOA and varied as a function of precipitation manipulations, AOA still contributed up to 37% of the total NO emissions from *Control* soils and were a persistent source of NO across the precipitation manipulations. AOA are known to tolerate dry conditions (Delgado‐Baquerizo et al. 2013, 2016) and maintain active nitrification even when N availability is low (Prosser et al. 2019). However, AOA are only thought to dominate ammonia‐oxidizing activity when they are much more abundant than AOB (i.e., AOA:AOB > 10) (Prosser and Nicol 2012), which was not the case at our site (Figure 6C). Furthermore, because AOA may be more efficient nitrifiers relative to AOB (Mushinski et al. 2019; Prosser et al. 2019), limiting the release of NO or N_2_O to the atmosphere, it was surprising that AOA still emitted 12%–37% of the NO measured from control soils in this study. Despite their limited ability to emit NO during nitrification, AOA could have still released nitrification intermediates into the soil environment (e.g., NH_2_OH and NO_2_^−^), favoring NO production via abiotic NH_2_OH decomposition, chemodenitrification, or microbial denitrification (Heil et al. 2016; Zhu‐Barker et al. 2015). The conversion of AOA nitrification intermediates to NO would still be classified as AOA‐derived NO emissions in our assays, likely explaining how AOA‐derived NO emissions persisted in our study. Thus, AOA‐driven nitrification may persist even through extreme shifts in precipitation, potentially becoming an increasingly important source of mineral N in drylands that are forecasted to become drier (Lewin et al. 2024).

Persistent AOA activity, together with sustained rates of net N mineralization and nitrification measured across our lab incubations and projected decreases in soil moisture throughout the southwestern United States (Dai 2013; Bradford et al. 2020), raise the possibility for NO_3_^−^ to accumulate in drying soils and produce N_2_O via denitrification when dry soils wet up. When we incubated the soils that were exposed to extreme moisture stress (*Winter‐*) at 100% WHC, we measured substantial increases in N_2_O emissions that were positively correlated with soil extractable NO_3_^−^ (Figure 3B), likely because of lower plant N uptake given reductions in plant biomass (Spasojevic et al. 2022). When these dry soils wet up, microbes may initially outcompete drought‐stressed plants for N (Liu et al. 2020), allowing denitrifiers to reduce NO_3_^−^ to N_2_O. Plant death in the water‐stressed *Winter‐*plots may have also made more C bioavailable (Slessarev et al. 2020), which can stimulate microbial O_2_ consumption during decomposition and favor denitrification (Rotkin‐Ellman et al. 2004; Schlüter et al. 2024), further amplifying N_2_O emissions and helping to explain why we measured negative net N mineralization and nitrification rates in the lab (Figure 5A,B)—N losses via N_2_O emission would have lowered soil N pools during the incubation. Our findings are consistent with studies showing that denitrifiers can persist during periods of extreme drought and heat to emit N_2_O when soils are wetted (Harris et al. 2021; Krichels et al. 2023a). Over longer time scales, prolonged dry periods may also slow N inputs to the soil by decreasing plant growth and N‐fixation by biocrust communities (Belnap and Lange 2003). If N inputs from plants and biocrusts are lowered under prolonged drought, they may be unable to replenish N losses from denitrification, contributing to ecosystem N limitation. Taken together, shifts in precipitation that are extreme enough to reduce plant cover may have consequences on the emission of N_2_O, an important greenhouse gas and N loss pathway in soils.

## 5 Conclusions

Using a field precipitation manipulation experiment combined with soil laboratory incubations, we show that both increasing and decreasing summer precipitation amounts can favor AOB‐derived NO emissions when soils wet up at the end of the summer, a period often characterized by substantial gaseous N losses across dryland ecosystems (Homyak et al. 2014; Osborne et al. 2022). Moreover, we show that inducing more severe water limitation by excluding winter precipitation may push dryland ecosystems across aridity tipping points beyond which AOB are not stimulated by excess soil N availability, but AOA contributions to NO emissions persist (Elrys et al. 2024). The consequences of crossing this aridity tipping point became evident in the *Winter‐* treatment, where NO_3_^−^ accumulated in soils and led to high N losses via the emission of N_2_O (a powerful greenhouse gas) upon rewetting. Expected increases in precipitation variability (Polade et al. 2017), along with projected decreases in soil moisture (Dai 2013; Bradford et al. 2020), may, therefore, influence N gas emissions from Pinyon‐Juniper drylands according to the magnitude and season during which the changes in precipitation occur.

## Author Contributions

**Sharon Zhao:** data curation, formal analysis, investigation, writing – original draft, writing – review and editing. **Alexander H. Krichels:** conceptualization, data curation, formal analysis, investigation, methodology, supervision, validation, visualization, writing – review and editing. **Elizah Z. Stephens:** conceptualization, data curation, formal analysis, investigation, methodology, supervision, validation, visualization, writing – review and editing. **Anthony D. Calma:** data curation, investigation, writing – review and editing. **Emma L. Aronson:** conceptualization, funding acquisition, writing – review and editing. **G. Darrel Jenerette:** conceptualization, investigation, methodology, project administration, writing – review and editing. **Marko J. Spasojevic:** conceptualization, data curation, investigation, methodology, project administration, validation, visualization, writing – review and editing. **Joshua P. Schimel:** conceptualization, formal analysis, funding acquisition, methodology, project administration, validation, visualization, writing – review and editing. **Erin J. Hanan:** formal analysis, validation, writing – review and editing. **Peter M. Homyak:** conceptualization, formal analysis, funding acquisition, investigation, methodology, project administration, supervision, validation, visualization, writing – review and editing.

## Conflicts of Interest

The authors declare no conflicts of interest.

## Supporting information


Data S1.

## Data Availability

The data that support the findings of this study are openly available on Dryad, “Data from: Nitrogen availability and changes in precipitation alter microbially‐mediated N emissions from a Pinyon Juniper dryland” at https://doi.org/10.5061/dryad.mpg4f4r72.
