# Supplementary material for: Nitrogen Availability and Changes in Precipitation Alter Microbially Mediated NO and N2O Emissions From a Pinyon–Juniper Dryland
Source: Glob Chang Biol. 2025 Mar 27;31(3):e70159. doi: 10.1111/gcb.70159 (PMC11948459; doi:10.1111/gcb.70159)
Supplement: Supplementary file 1 — Data S1. [file GCB-31-e70159-s001.docx]

Supplementary Material for

**Nitrogen availability and changes in precipitation alter microbially-mediated N emissions from a Pinyon Juniper dryland**

Sharon Zhao^1*^, Alexander H. Krichels^1,2^, Elizah Z. Stephens^1^, Anthony Calma^1^, Emma L. Aronson^3^, G. Darrel Jenerette^4^, Marko J. Spasojevic^5,6^, Joshua P. Schimel^7^_,_ Erin J. Hanan^8^, Peter M. Homyak^1^

^1^Department of Environmental Sciences, University of California, Riverside, CA

^2^USDA Forest Service, Rocky Mountain Research Station, Albuquerque, NM

^3^Department of Microbiology and Plant Pathology, University of California, Riverside, CA

^4^Department of Botany and plant Sciences, University of California, Riverside, CA

^5^Department of Evolution, Ecology, and Organismal Biology, University of California, Riverside, CA

^6^Environmental Dynamics and GeoEcology Institute, University of California Riverside, CA 92521, USA

^7^Department of Ecology, Evolution and Marine Biology, University of California, Santa Barbara, CA

^8^Department of Natural Resources & Environmental Science, University of Nevada, Reno, NV

*Corresponding author email: [szhao017@ucr.edu](mailto:szhao017@ucr.edu)

**Contents of this file**

Table S1

Figures: S1, S2, S3, S4, S5, S6, S7

**Table S1:** Seasonal precipitation exclusion and water addition amounts from 2018 – 2021. Precipitation amounts (mm) are calculated from the Pinyon Crest Weather Station. In Winter 2021, no water was collected due to leaks in the water collection system; groundwater from a local well was used to irrigate the *Winter+* plots.

| Season + Year | Added Water (mm) | Excluded Rainfall (mm) | Total Annual Rainfall (mm) |
| --- | --- | --- | --- |
| Summer 2018 | 117 | 90 | 194 |
| Winter 2018-2019 | 58 | 335 |  |
|  |  |  |  |
| Summer 2019 | 22 | 73 | 400 |
| Winter 2019-2020 | 132 | 252 |  |
|  |  |  |  |
| Summer 2020 | 0 | 0 | 189 |
| Winter 2020-2021 | 106 | 85 |  |
|  |  |  |  |
| Summer 2021 | 15 | 58 | 185 |
| Winter 2021-2022 | 7 | 111 |  |


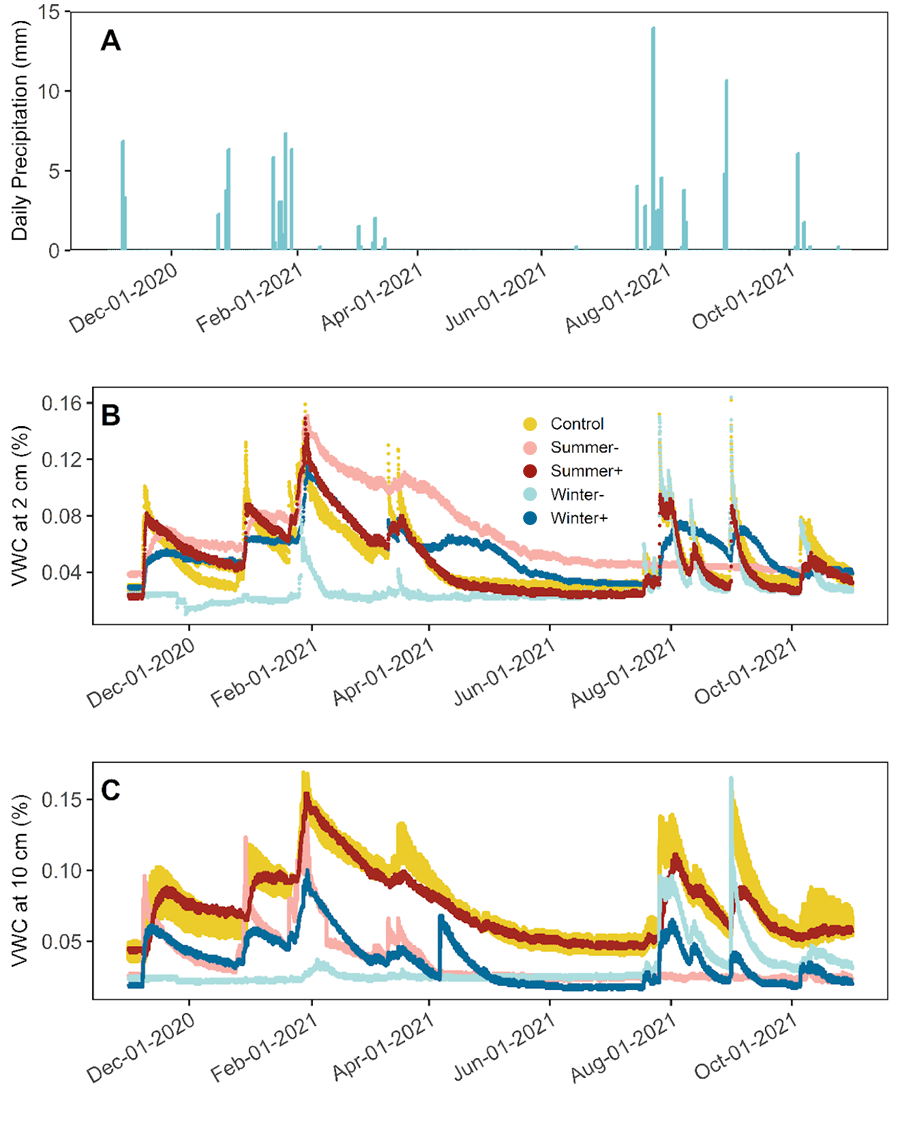


**Figure S1.** A) Daily precipitation and B) hourly volumetric soil water content (VWC) at 2 cm and C) 10 cm depth measured over the one-year period prior to collecting soils for the lab experiment. For the VWC data, one sensor was installed at each of the two depths within each of the measured plots. Moisture sensors were installed in one plot within each treatment as well as in two of the *Control* plots. Summer precipitation was excluded from June through October and winter precipitation was excluded for the remainder of the year.


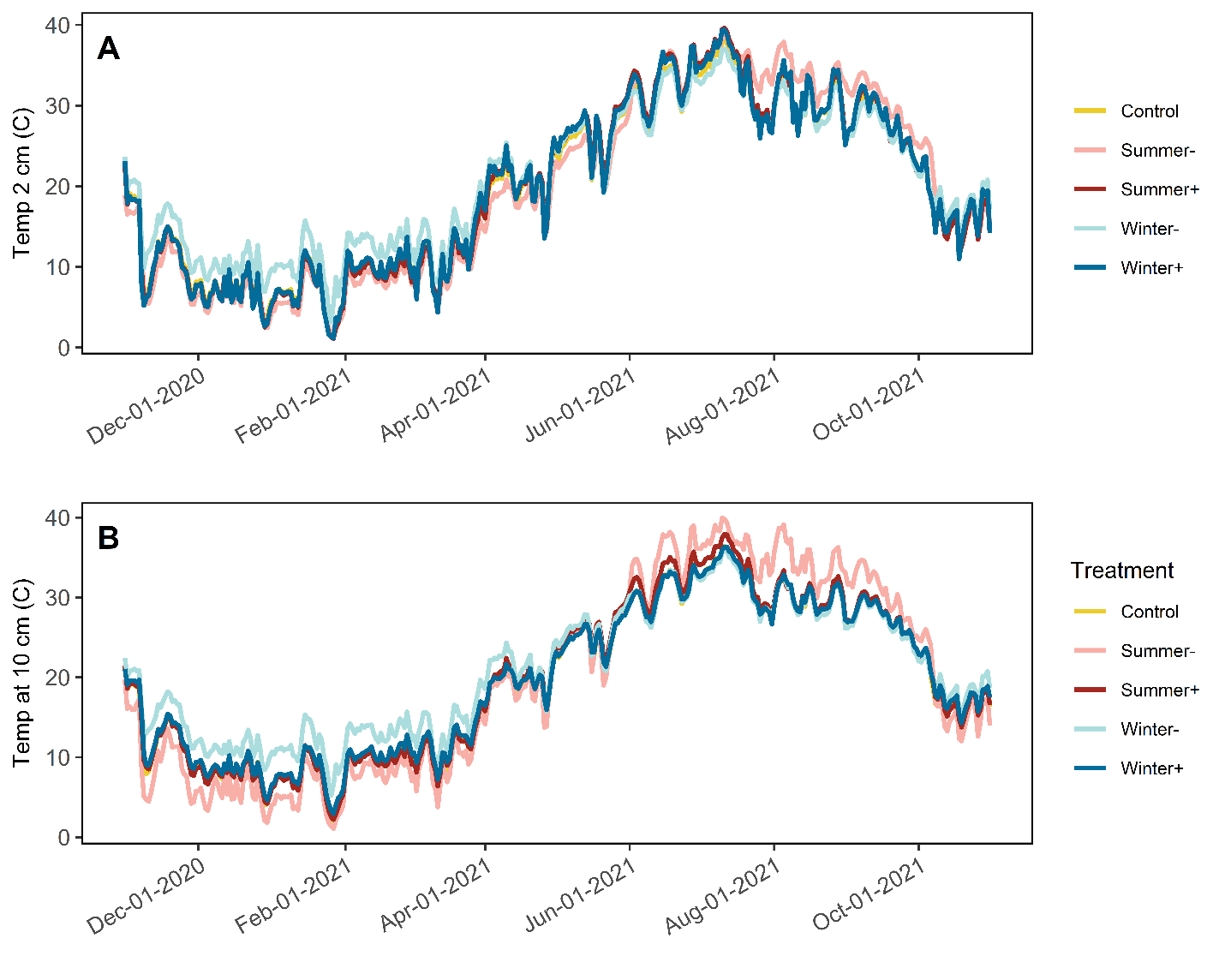


**Figure S2.** A) Average daily temperature at 2 cm and B) 10 cm depth measured over the one-year period prior to collecting soils for the lab experiment. One sensor was installed at each depth within each of the measured plots. Temperature sensors were installed in one plot within each treatment as well as in two of the *Control* plots. Summer precipitation was excluded from June through October and winter precipitation was excluded for the remainer of the year.


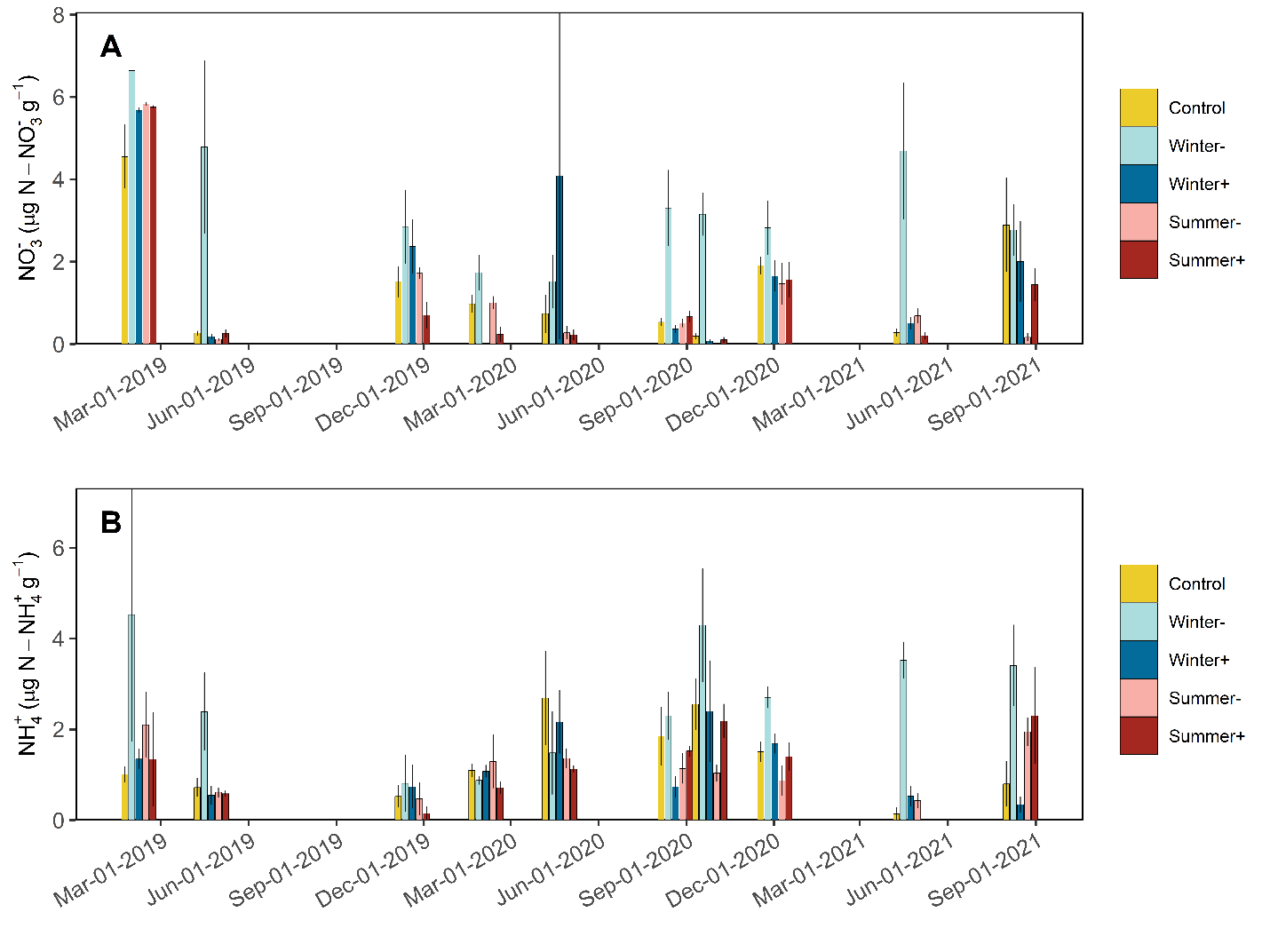


**Figure S3:** Soil extractable A) nitrate (NO_3_^-^) and B) ammonium (NH_4_^+^) concentrations from field-collected soils between 2019 and 2021. Bars represent the mean and error bars represent standard errors (n = 4, except for control treatments where n = 8).


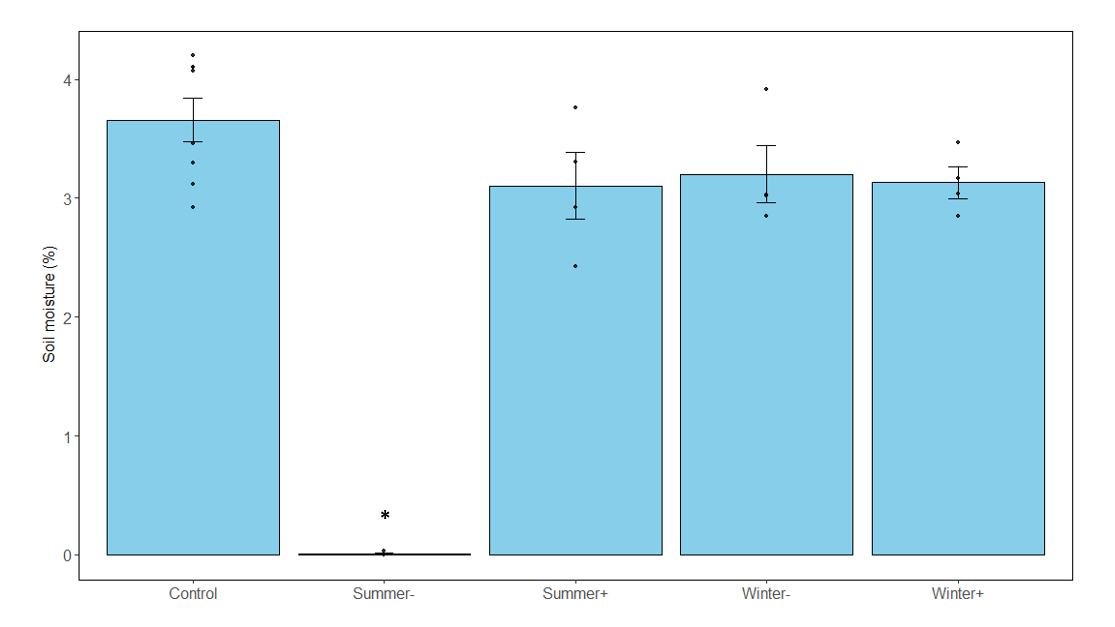
**Figure S4:** Gravimetric soil water content (%) from the field at the time of sample collection. The asterisk indicates the *Summer-* treatment is significantly different from all other treatments (p < 0.05). Error bars represent the standard error of each field (n = 4, except for control treatments where n = 8).

**
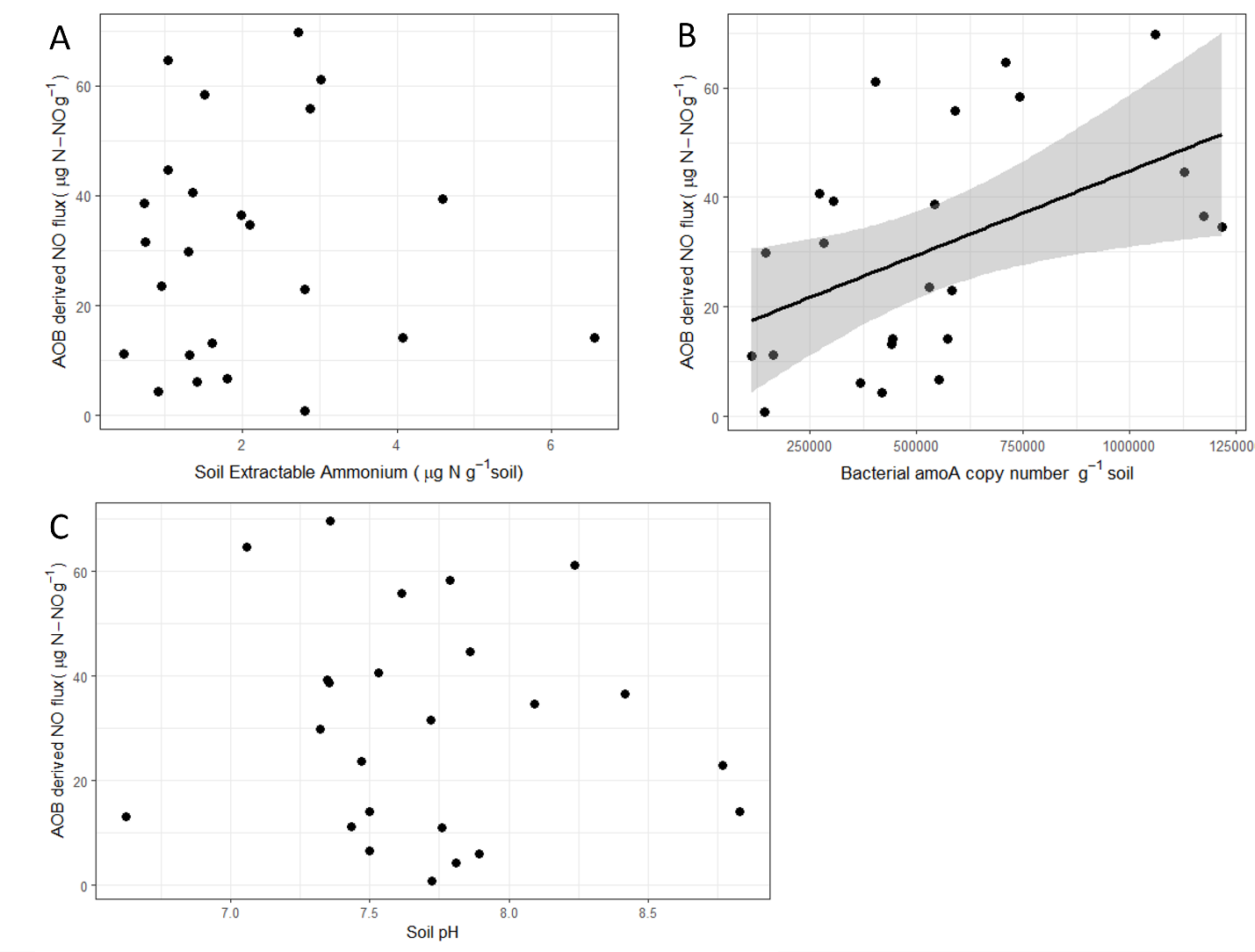
**

**Figure S5:** Regression plots for AOB-derived NO fluxes (Y-axis) and dependent variables (X-axis). Significant linear relationships (*p* < 0.05) are shown with a black line. For B., the equation of the line, y = 0.00003087x + 13.98 with a p-value = 0.0165.

**
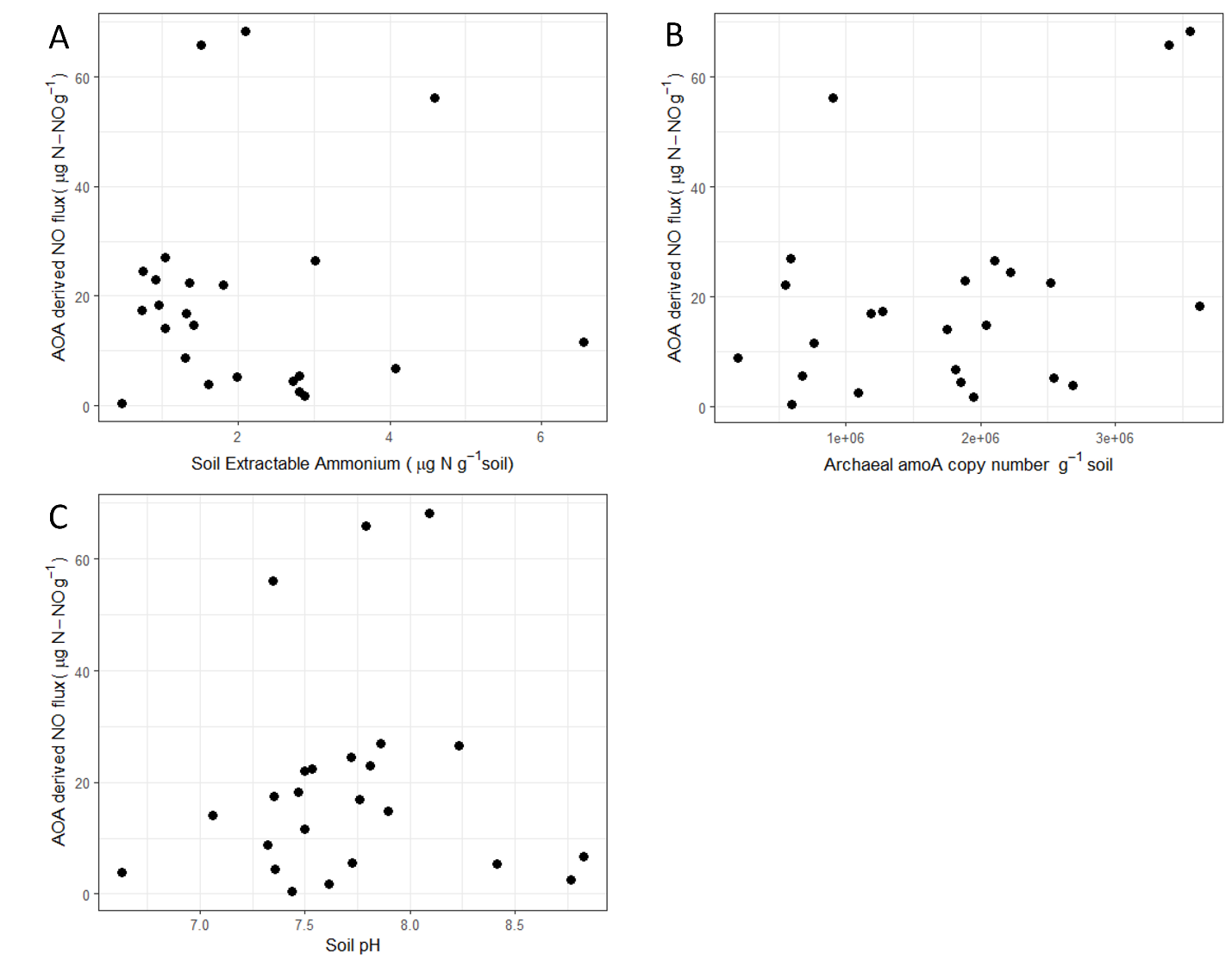
**

**Figure S6:** Regression plots for AOA-derived NO fluxes (Y-axis) and dependent variables (X-axis). Significant linear relationships were not detected (*p* < 0.05).

**
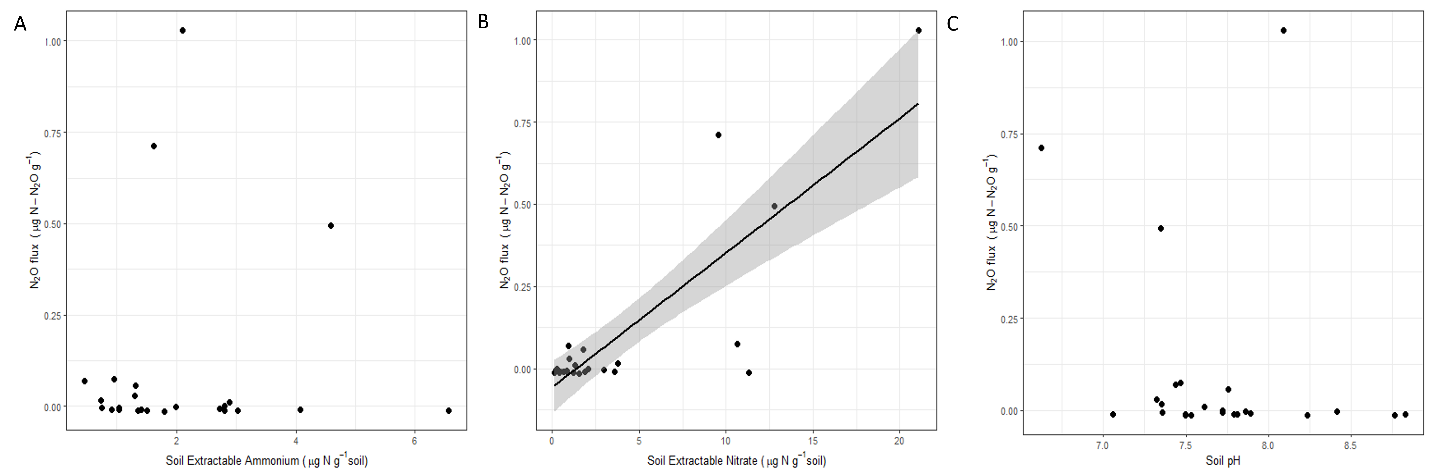

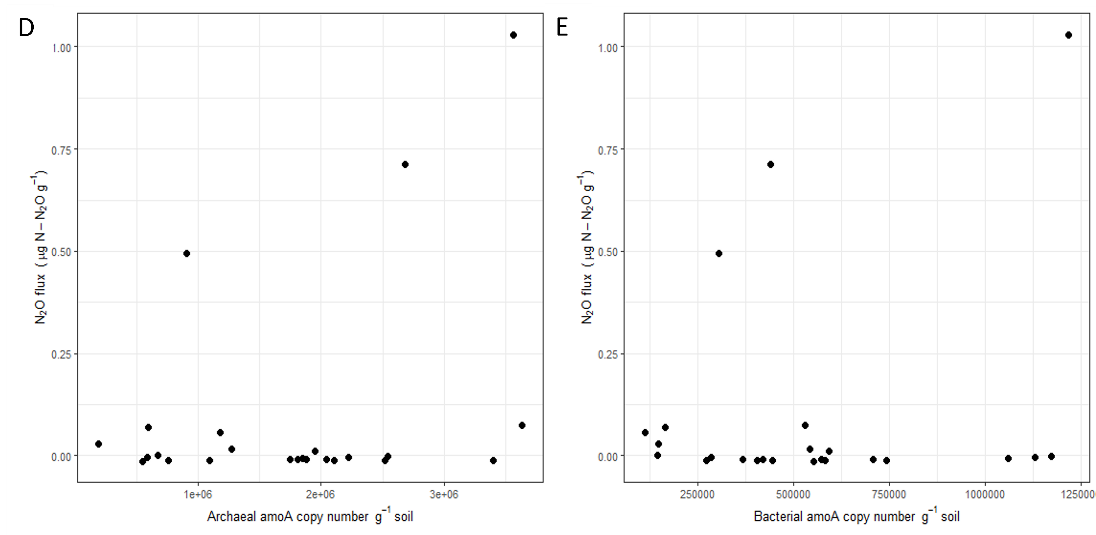
**

**Figure S7:** Regression plots for N_2_O emissions derived from soils held at 100% WHC (Y-axis) and dependent variables (X-axis). Significant linear relationships (*p* < 0.05) are shown with a black line. For B., the equation of the line, y = 0.040914x – 0.056905 with a p-value = 6.58e-7.
